# Functional Outcome of Hemiarthroplasty of the Hip for Unstable Intertrochanteric Fractures of the Femur in Elderly Patients: A Prospective Study

**DOI:** 10.7759/cureus.32526

**Published:** 2022-12-14

**Authors:** Jasveer Singh, Dinesh Kumar, Sunil Kumar, Ankit Mittal, Pradeep Kumar Gupta, Ajay Kumar Rajput, Ravi Kant, Santosh Kumar Singh

**Affiliations:** 1 Department of Orthopaedics, Uttar Pradesh University of Medical Sciences, Etawah, IND; 2 Department of Orthopaedics and Traumatology, Employee's State Insurance Corporation Hospital, Jajmau, Kanpur, IND

**Keywords:** bipolar, complications, cement, unstable intertrochanteric fracture, hemiarthroplasty

## Abstract

Introduction

It is frequently challenging to treat an unstable intertrochanteric fracture in elderly people by internal fixation because of difficult anatomical reduction, poor bone quality, the need for prolonged bed rest and restricted ambulation. As an alternative to internal fixation, cemented bipolar hemiarthroplasty has been used as a treatment for unstable intertrochanteric fractures to avoid the postoperative immobilization-related complications. The aim of this study was to evaluate the functional and clinical outcomes of primary cemented bipolar hemiarthroplasty for elderly patients with unstable intertrochanteric fractures.

Methodology

A prospective study was conducted that included 30 patients who were admitted to the apex trauma center at a tertiary care center from January 2019 to August 2020 with unstable intertrochanteric fractures (Association for Osteosynthesis/Orthopaedic Trauma Association, or AO/OTA, types 31-A2.2 and 31-A2.3); patients treated with cemented bipolar hemiarthroplasty, with at least one year of follow-up were included in the study. Basic descriptive statistics were used and the results were presented in frequencies, percentages for categorical variables and means and standard deviations for continuous variables.

Results

According to the Harris Hip Score, at the end of 12 months, 9 patients (30%) had excellent results, 14 patients (46.67%) had good results, 5 patients (16.67) had fair results, and 2 cases (6.67) had poor results. With cemented hemiarthroplasty, 87.7% of older patients with unstable intertrochanteric fractures were able to walk sooner, and the results were good.

Conclusion

Hemiarthroplasty of the hip with a cemented bipolar prosthesis appears to be a reliable treatment method for the management of unstable intertrochanteric fractures in elderly patients with osteoporosis; it allows for early ambulation and leads to a favorable functional outcome in most patients following surgery.

## Introduction

A hip fracture represents a disturbing and potentially ominous landmark in a person's health history [1]. An intertrochanteric fracture is one of the most common health problems in the elderly, having a high one-year mortality rate of up to 20% [2,3]. An increase in life expectancy and a sedentary lifestyle increased the incidence of these fractures from 1.66 million in 1990 to an expected 6.26 million by 2050 [4]. These fractures occur in the elderly as a result of trivial trauma, most commonly, sideways falls from a standing height [5]. One of the major risk factors for these fractures is osteoporosis, and females are more likely to be affected than males [4,5].

The management of intertrochanteric fractures is done by two modes: conservative and operative. At the moment, the conservative method has a limited role and is used only in patients who are at a high risk of anesthesia and surgery, as well as non-ambulatory patients who have minimal pain after fracture. Successful surgery of an unstable intertrochanteric fracture should provide a stable, pain-free hip with a good range of movement. Osteosynthesis of these fractures with an angled blade plate, dynamic hip screw, and cephalomedullary nailing in the osteoporotic bone has a number of problems, such as unstable geometry of the fracture that is hard to fix, pullout of screws, poor screw purchase, varus collapse of the fracture, and slow rate of union, which can cause a decubitus ulcer, an upper respiratory tract infection, or pneumonia if the patient stays in bed for months [6-8].

Bipolar hemiarthroplasty emerges as a good implant for unstable intertrochanteric fractures to overcome these shortcomings by bypassing the stages of bone healing [9]. It allows early mobilization, fewer hospital stays, and a good range of motion [8-11]. It can be done primarily or after the failure of conservative or internal fixation [10-12]. The aim of this study was to prospectively evaluate the functional and clinical outcomes of primary cemented bipolar hemiarthroplasty for older patients with unstable osteoporotic intertrochanteric fractures (Association for Osteosynthesis/Orthopaedic Trauma Association, or AO/OTA, types 31-A2.2 and 31-A2.3) [13].

## Materials and methods

Source of data

Data was collected from 30 elderly patients admitted and managed at the Department of Orthopaedics (Uttar Pradesh University of Medical Sciences, Saifai, Uttar Pradesh) from January 2019 to August 2020 with cemented bipolar hemiarthroplasty who had an unstable intertrochanteric fracture (AO/OTA types 31-A2.2 and 31-A2.3) and met the inclusion criteria, with at least one year of follow-up. Proper institutional ethics committee clearance (1364/UPUMS/Dean/M/Ethical/183/2018) was obtained prior to the start of the study and written informed consent was taken from all the patients.

Inclusion/exclusion criteria

Patients over 60 years of age, those who were independently ambulatory before sustaining the fracture, and who had unstable intertrochanteric fractures (AO/OTA types 31-A2.2 and 31-A2.3) with osteoporosis (Singh Index, or SI, ≤3) were included in the study. Patients who were not willing to participate in the study or were unfit for surgery, those with distal neurovascular compromise and neuropathy, with open or stable intertrochanteric fractures, pathological fractures, preexisting hip infection or polytrauma, and those who were lost to follow up before one year were excluded.

Preoperative evaluation

A detailed history of the mode of injury and comorbidities was taken. Patients were clinically evaluated for fracture evidence such as limb attitude, loss of transmitted movement, and limb shortening. Standard good quality orthogonal antero-posterior and lateral radiographs of the fractured extremity were taken. Fractures were classified according to the AO/OTA classification. The Singh Index, which is based on the trabecular pattern of the proximal femur and categorizes osteoporosis into six grades, was used as an evaluation tool for detecting osteoporosis with a standard digital X-ray scan of the pelvis in the supine position [14,15]. Grade I and grade VI correspond to severe osteoporosis and normal bone density, respectively. Traction was applied to all patients. Blood investigations were done. After a pre-anesthetic checkup and fitness for surgery, the patient was scheduled for operative management.

Surgical procedure

**Figure 1:**
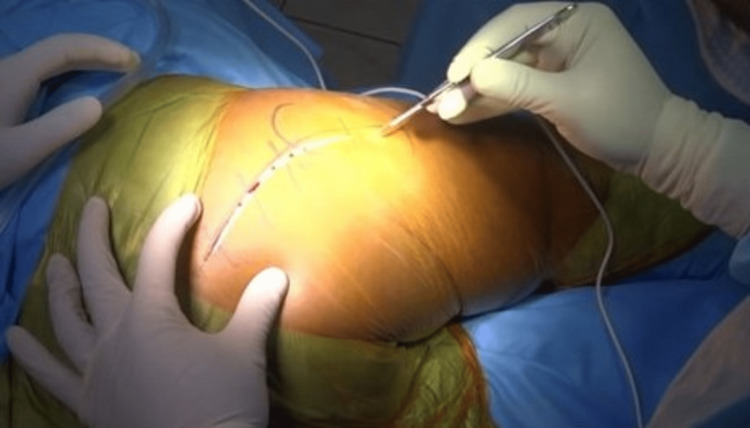
Lateral decubitus position of the patient and exposure of the hip by the Southern (posterior) approach

All surgeries were carried out in the elective operation room using all aseptic precautions by the team of three experienced surgeons. All surgeries were performed under spinal anesthesia with the patient lying on the unaffected side in a lateral decubitus position. Exposure of the hip was done by the Southern (posterior) approach (Figure 1).

**Figure 2:**
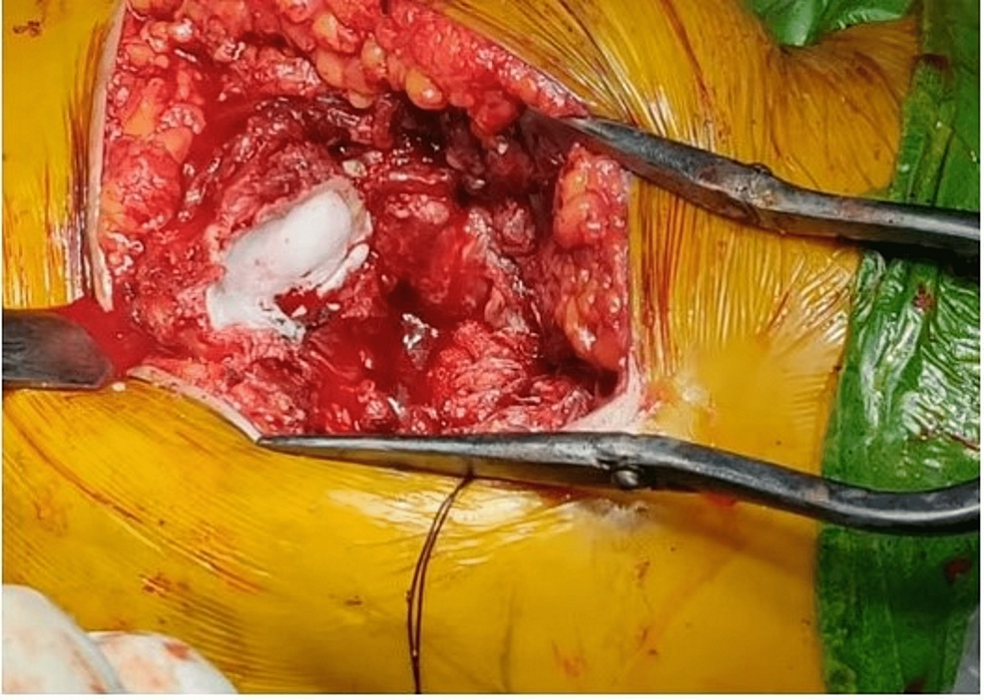
Reconstruction of the calcar by cement

An incision was made 6-8 cm above and posterior to the posterior aspect of the greater trochanter and running in line with the fibers of the gluteus-maximus. Rotators were identified and tagged. The capsule of the hip was exposed and a capsulotomy was performed. The femoral head was removed and measured in size. The acetabulum was prepared, the remnant ligamentum teres was completely excised and the remaining soft tissue from the pulvinar region was curetted. Femoral stem preparation was done with reamers and broaches of appropriate size. The reconstruction of the trochanter was done with the stainless steel (SS) wire and Ethibond sutures as required. Second-generation cementing techniques with a distal cement restrictor were used to cement the femoral stem. In the final step, the insertion of the stem of the prosthesis was sunk to the point on the stem that was marked previously to equalize limb length. Calcar reconstruction was done in all the patients with the help of cement (Figure 2).

After hardening of the cement, the greater trochanter and calcar were retightened with the SS wire. Closure was done in layers under a negative suction drain.

Postoperative follow-up

Postoperatively, antibiotics and anti-inflammatory analgesics were administered. The negative suction drain was removed after 48 hours. The routine postoperative physiotherapy protocol along with quadriceps muscle-strengthening exercises was started one day after surgery. Patients were made to sit up on the second day, stand up with support (walker) on the third day, and were allowed full weight bearing and to walk with the help of a walker on the fourth postoperative day, depending on their pain tolerance; they were encouraged to walk thereafter. Sitting cross-legged and squatting were not allowed. Suture removal was done on the 14th postoperative day. Patients were followed up at an interval of 6 weeks, 3 months, 6 months, and 12 months, and thereafter annually. All the patients were analysed clinically and radiologically at each follow-up.

The Harris Hip Scores, which classify scores below 70 as bad, 70-79 as fair, 80-89 as good, and 90-100 as excellent, were used to evaluate the clinical evaluation [16]. Any postoperative complications were also evaluated at each follow-up. Standard anteroposterior and lateral X-ray views were taken at each follow-up to evaluate the loosening of the prosthesis, malpositioning of the stem, and sinking of the stem.

Statistical analysis

Basic descriptive statistics were used and the results were presented in frequencies, percentages for categorical variables and means and standard deviations for continuous variables. IBM SPSS Statistics for Windows, Version 24.0 (IBM Corp., Armonk, NY) was used for all data analysis.

## Results

All patients above 60 years of age were included. In our study, patients' age ranged from 62 to 84 years. The average age of the study population was 73.7 ± 5.25 years, and the female-to-male ratio was 3:2. The fact that most of the people in this study were women might be because osteoporosis and weak bone mass are more common in women after menopause. The left side extremity was involved in 16 patients (53.33%).

Fifty percent of patients sustained road traffic accidents, 30% had a history of falls from a height, and 20% sustained minor trauma due to falls on the ground while walking that resulted in fractures. This again suggests minimal trivial trauma causes most of the intertrochanteric fractures in elderly people due to weak bone mass. According to the AO/OTA classification for trochanteric region fractures, 19 patients presented with type 31-A2.2 fractures and 11 patients with type 31-A2.3 fractures. According to the SI, grade III osteoporosis was the most common, seen in 16 patients (53.33%); 11 patients (36.67%) had grade II and three patients (10%) had grade I osteoporosis.

Many patients had age-related comorbidities. Hypertension was seen in nine patients and diabetes mellitus in seven patients, while four patients were found to be suffering from both conditions, and they were managed accordingly. Six patients presented with anemia, which was treated by blood transfusion preoperatively as per the recommended protocol. Five patients were suffering from asthma, while three patients had chronic obstructive pulmonary disease (COPD). One patient had chronic kidney disease for which he was on dialysis. One patient had a history of ischemic heart disease, for which he was on regular medication. All the patients were operated on after getting fit for surgery. The average time interval from admission to operation was 6.73 ± 1.48 days. Sociodemographic characteristics of patients included are depicted in Table 1.

**Table 1:** Sociodemographic details of patients ^a^Data is presented as mean ± standard deviation. RTA: road traffic accident, AO/OTA: Association for Osteosynthesis/Orthopaedics Trauma Association, COPD: chronic obstructive pulmonary disease

S.no.	Variables		Frequency (n)	Percentage
1	Age(years)^a^		73.7 ± 5.25	
2	Sex	Male	12	40
		Female	18	60
3	Side involved	Right	14	46.67
		Left	16	53.33
4	Mode of injury	RTA	15	50
		Fall from a height	9	30
		Fall while walking	6	20
5	Type of fracture	AO/OTA type 31-A2.2	19	63.33
		AO/OTA type 31-A2.3	11	36.67
6	Grading of osteoporosis (Singh index)	Grade I	3	10
		Grade II	11	36.67
		Grade III	16	53.33
7	Associated comorbidities	Diabetes mellitus	7	23.33
		Anaemia	6	20
		Hypertension	9	30
		Chronic kidney disease	1	3.33
		Asthma	5	16.67
		COPD	3	10
		Ischemic heart disease	1	3.33
8	Interval between the injury and surgery (days)^a^		6.73 ± 1.48	

The average duration of surgery was 78.5 ± 15.1 minutes. The average intraoperative blood loss during the surgery was 353 ± 41.6 ml. Postoperative single unit blood transfusion was required in 17 (56.67%) patients. The first postoperative surgical dressing was done on the third day, and the drain was removed. Patients were encouraged to fully weight bear on the third day of surgery. Initially, they were advised to walk with a walker and later switch to a stick for support. Most patients could walk without support after six days of surgery. We discharged patients following suture removal on the 14th postoperative day, averaging 15.1 ± 1.12 days in the hospital. The femoral stem was positioned centrally along the longitudinal axis of the femoral shaft in 26 patients. Six patients had valgus stem positioning, whereas one patient had varus stem positioning.

Postoperatively, four patients had superficial infection at the incision site and two patients presented with superficial bed sores; both the patients were managed with intravenous antibiotics and surgical dressings. One patient developed upper respiratory tract infection, which was managed by intravenous antibiotics. The functional outcome was graded according to the Harris Hip Score. In our study, at the end of 12 months, nine patients had excellent, 14 had good, three had fair, and four had a poor clinical score. Intraoperative and postoperative variables of the patients are summarized in Table 2.

**Table 2:** Intraoperative and postoperative variables ^a^Data is presented as mean ± standard deviation.

S.no.	Variables		Frequency (n)	Percentage
1	Operating time (minutes)^a^		78.5 ± 15.1	
2	Intraoperative blood loss (ml)^a^		353 ± 41.6	
3	Need of postoperative blood transfusion		17	56.67
4	Duration of stay in the hospital (days)^a^		15.1 ± 1.12	
5	Time to full weight bearing after surgery (days)^a^		5.36 ± 1.49	
6	Femoral stem positioning	Central	26	86.67
		Valgus	3	10
		Varus	1	3.33
7	Postoperative complication	Superficial infection	4	13.33
		Superficial bed sore	2	6.67
		Upper respiratory tract infection	1	3.33
		Nonunion of greater trochanter	1	3.33
		Limb length discrepancy	11	36.67
8	Functional outcome (Harris Hip Score at the 12-month follow-up)	Excellent	9	30
		Good	14	46.67
		Fair	3	10
		Poor	4	13.33

A limb length discrepancy was observed in 11 patients (36.67%) postoperatively, out of whom, 7 patients (23.33%) had a shortening of less than 2 cm, which was very well compensated by the shoe rise. Two patients (6.67%) had a shortening of more than 2 cm, but they could walk well with the support of a stick. Two patients had lengthening of less than 1.5 cm (6.67%). One patient presented with nonunion of the greater trochanter due to SS-wire breakage, with no obvious limb pain under normal load. In our study, there was no incidence of sciatic nerve palsy, deep vein thrombosis, heterotopic ossification, postoperative dislocation of the prosthesis, loosening of the prosthesis, subsidence of the femoral stem, periprosthetic fracture, or acetabular erosion radiologically on follow-up. There was no need of revision surgeries in any case. There was no mortality recorded from the time of hospitalization to the one-year follow-up of the surgeries.

**Figure 3:**
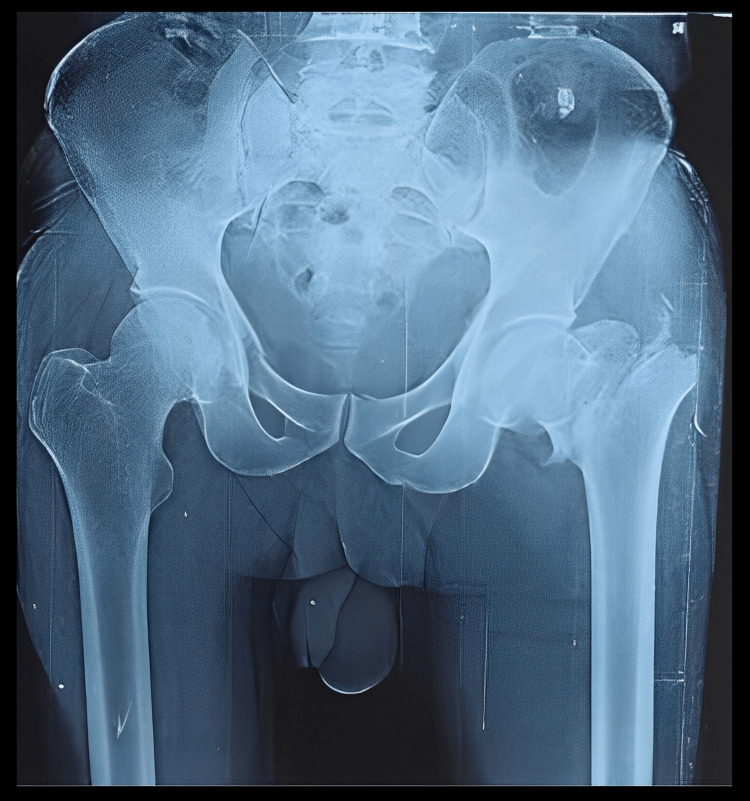
Anteroposterior X-ray view of the pelvis with both hip joints of a 66-year-old male showing an unstable intertrochanteric fracture of AO/OTA type 31-A2.2 of the left side AO/OTA type: Association for Osteosynthesis/Orthopaedics Trauma Association

Figure 3 shows the preoperative anteroposterior X-ray view of the pelvis with both hips of a 66-year-old male who sustained a post-traumatic unstable intertrochanteric fracture of AO/OTA type 31-A2.2 of the left side.

**Figure 4:**
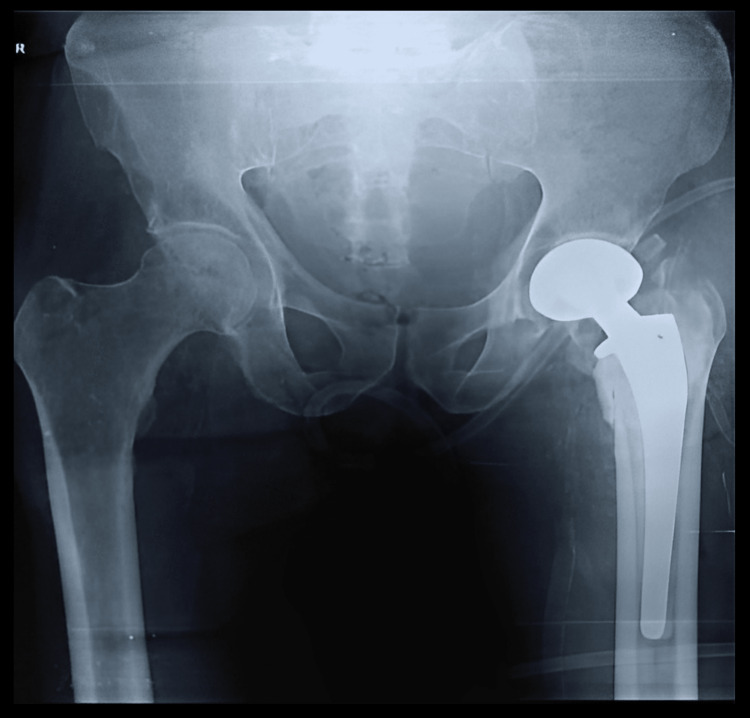
Postoperative anteroposterior X-ray view of the same patient managed by primary cemented bipolar hemiarthroplasty

He was managed by primary cemented bipolar hemiarthroplasty as shown in Figure 4.

**Figure 5:**
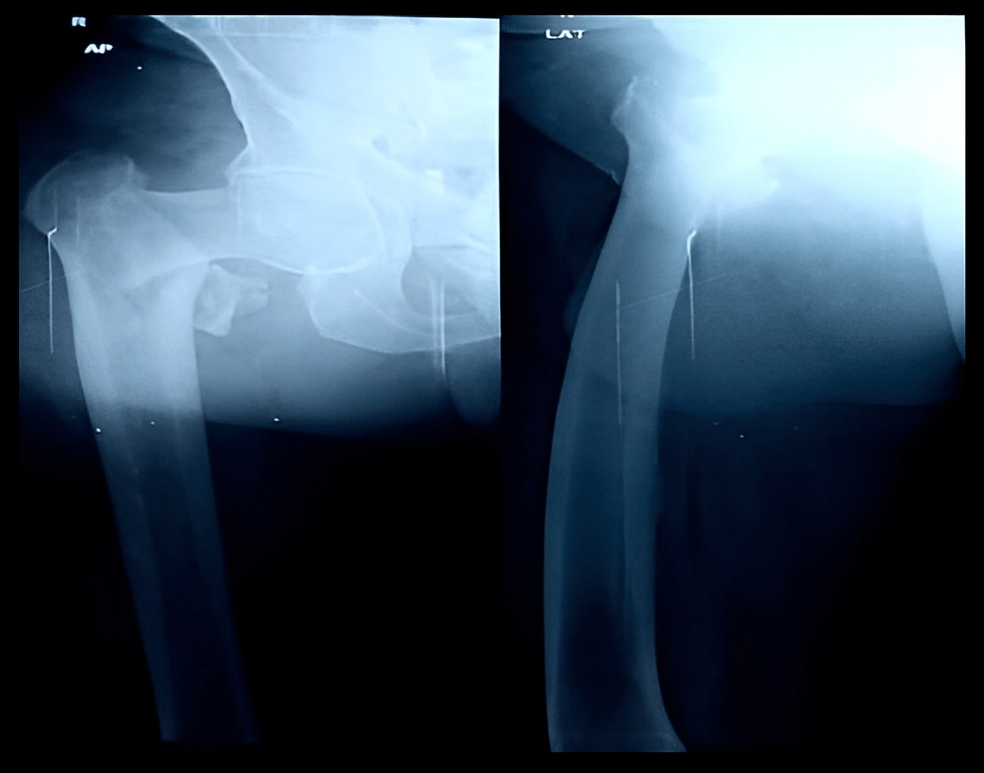
Anteroposterior X-ray view of a 70-year-old male showing an unstable intertrochanteric fracture of AO/OTA type 31-A2.3 of the right side AO/OTA type: Association for Osteosynthesis/Orthopaedics Trauma Association

Figure 5 shows the X-ray of the anteroposterior view in a 70-year-old male showing an unstable intertrochanteric fracture of AO/OTA type 31-A2.3.

**Figure 6:**
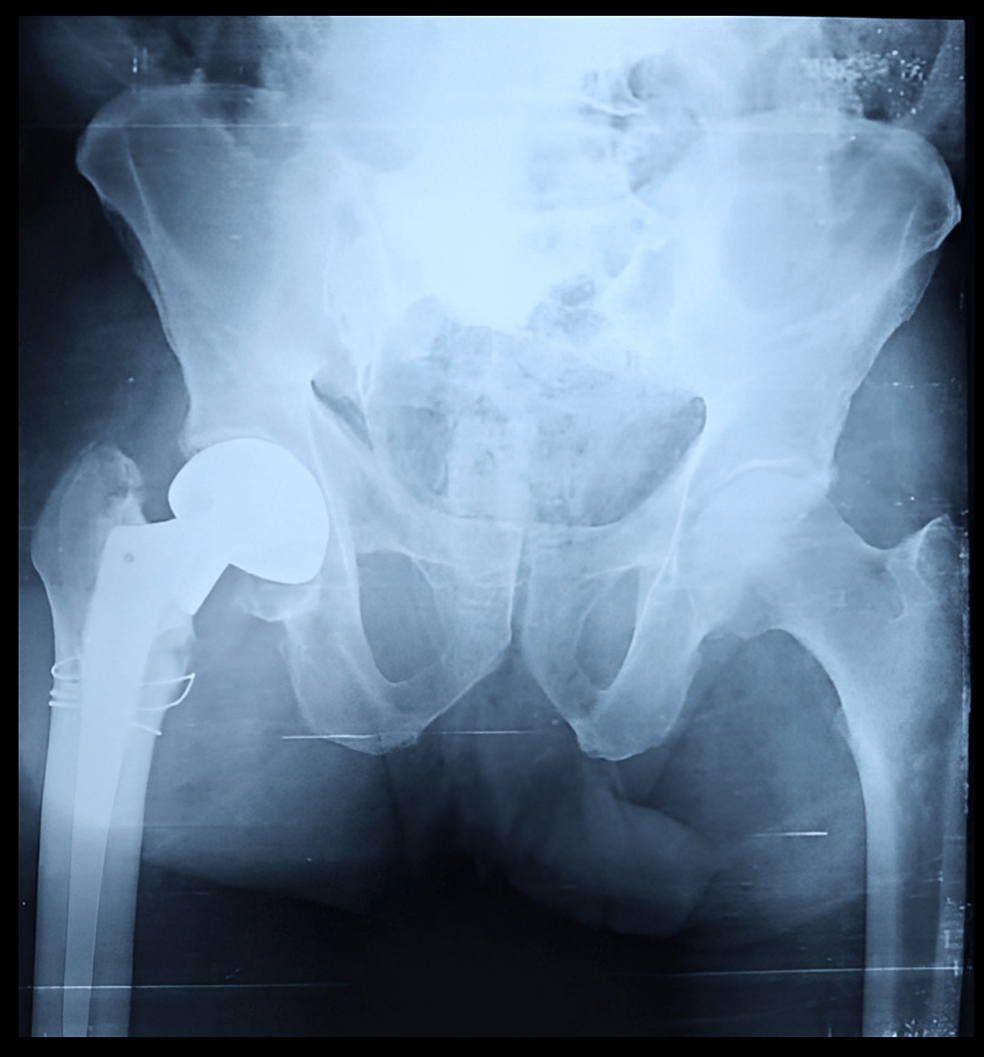
Postoperative X-ray of the same patient after cemented bipolar hemiarthroplasty along with posteromedial calcar reconstruction using cement and stainless steel wire

Figure 6 shows the postoperative X-ray after cemented bipolar hemiarthroplasty along with posteromedial calcar reconstruction using cement and SS wire.

## Discussion

Several surgical options are available for the treatment of unstable intertrochanteric fractures in osteoporotic elderly patients, but they still remain controversial. Due to weak, osteoporotic bones and the geometry of the fracture in these patients, it is hard to get a good grip of the screw in the femoral head. This leads to a high failure rate of internal fixation by proximal femoral nail (PFN) and sliding hip screw (SHS) and causes varus malposition, prolonged immobilization, and being bedridden for several weeks [11]. Although it is known that early ambulation following rigid fracture fixation may help reduce morbidity and mortality, determining the optimal treatment approach for these fracture types remains difficult. Primary cemented bipolar hemiarthroplasty has emerged as a better choice for treatment of unstable inter-trochanteric fractures and has been advocated with the view of making rehabilitation early and decreasing the incidence of complications of prolonged immobilization [17,18].

In our study, the average age of patients with unstable intertrochanteric fractures was 73.7 ± 5.25 years. This is in contrast to the older age group as reported by the western literature [10,12,17]. Our results are comparable with those of Rawate et al., who reported the average age as 79.35 years, Rodop et al. who reported 75.6 years, and Sancheti et al. reporting 77.1 years as the average age [19-21]. In the present study, the female-to-male ratio was 3:2. There was a female sex preponderance seen in our study, which may be due to postmenopausal osteoporosis and lower peak bone mass. Female preponderance in the Indian perspective was also observed by Sancheti et al. and Patil et al. in their retrospective studies [21,22]. The most common associated comorbidity was hypertension in 30% of cases, followed by diabetes in 23.3% of cases. They were all treated accordingly. In the study by Sancheti et al., 14% of patients had high blood pressure and 10% had diabetes [21]. In our study, the average blood loss was 353 ml. Our results are comparable with those of Rawate et al. who reported 409.37 ml blood loss, Sancheti et al. who reported 350 ml blood loss and Patil et al. who reported 354.5 ml intraoperative average blood loss [19,21,22]. In our study, 56.57% of patients required postoperative blood transfusions, while Rawate et al. and Kiran Kumar et al. found 28.12% and 55% of patients needed perioperative transfusions, respectively [19,23]. The average operating time was estimated at 78.5 minutes. In the initial cases, our operating time was in the higher range. With experience, the operating time was reduced, which was comparable with results by Sancheti et al. who had an average of 77.1 minutes, Rawate et al. who had an average time of 82.53 minutes, and Sinno et al. with an average of 112 minutes [19,21,24].

In the present study, postoperatively, limb length discrepancy over the operated limb was seen in 11 patients. Seven out of 30 patients had a shortening of less than 2 cm, so they were given a heel raise. Two patients had a shortening of more than 2 cm; they had a slight limp and used the support of a stick while walking; one patient had a lengthening of less than 2 cm. Siwach et al. reported shortening of less than 5 mm in 64% of cases, while 28% of cases had limb lengthening of between 5 and 10 mm [25]. They noticed the shortening was due to excessive sinking of the prosthesis following weight bearing. Kiran Kumar et al. reported that 20% of cases had a shortening of less than 2 cm, 10% of cases had a shortening of more than 2 cm, and one patient had a lengthening of more than 1.5 cm [23]. In this study, the mean time of full weight bearing was 5.36 ± 1.49 days, while in the studies by Sancheti et al. and Kiran Kumar et al., the mean time was 4.2 and 5.4 days, respectively [21,23]. Eighteen patients were discharged on postoperative day 14 after suture removal. The mean number of days spent by the patient in the hospital was 15.1 ± 1.12 days, which is comparable to the outcome of other studies: Rawate et al., 14.53 days; Sancheti et al., 10.96 days; Kiran Kumar et al., 13.3 days [19,21,23].

In our study, at the end of 12 months, 9 patients (30%) had excellent results, 14 patients (46.67%) had good results, 5 patients (16.67) had fair results, and 2 cases (6.67) had poor results. Excellent to fair results were achieved in 93.3% of cases, which is comparable to other studies, as Sancheti et al., in a study of 35 patients treated with hemiarthroplasty, reported excellent to fair results in 91% patients according to the Harris Hip Score [21]. Kiran Kumar et al. achieved 90% excellent to fair results as assessed by the Harris Hip Score [23]. Saoudy et al. in a series of 30 cases reported 86% fair to excellent results (4 cases as excellent, 12 as good, 10 as fair, and 4 as poor) [26]. Similarly, Elmorsy et al. reported that the Harris Hip Score at the final follow-up ranged between 93 and 51, with a mean of 78.19, in which four cases (9.76%) were rated excellent, 16 (30.02%) were rated good, 16 (30.02%) were rated fair, and 5 (12.02%) were rated poor [27].

Hongku et al. conducted a systematic review to compare the efficacy of osteosynthesis (dynamic hip screw, proximal femoral nail) and bipolar hemiarthroplasty and showed that dynamic hip screw and PFN had a significantly higher risk of operative failure compared with bipolar hemiarthroplasty in unstable intertrochanteric fractures in elderly patients [28]. Chowdhury et al. did a systematic review to compare hemiarthroplasty and DHS fixation for intertrochanteric fractures in elderly patients [29]. They found that at 12 months, hemiarthroplasty led to significantly higher Harris Hip Scores and allowed patients to start bearing weight sooner than with DHS fixation.

In a study comparing total hip replacement arthroplasty and cemented bipolar hemiarthroplasty, Fan et al. found that there was no difference between total hip replacement and cemented bipolar hemiarthroplasty in functional outcomes and managing the pain [30]. However, they also noted that there was no evident difference in the hospitalization period, general complications, and rate of revision and mortality during the follow-up. They also concluded that total hip replacement arthroplasty posed some unique challenges for geriatric patients, including higher intraoperative blood loss, longer duration of surgery, increased rates of dislocation, impaired reflexes, and cognitive decline, and greater costs, suggesting that hemiarthroplasty might be a better or more reasonable choice for unstable intertrochanteric fractures of the femur in elderly patients.

Limitations

Due to the limited sample size and short follow-up, the current study was unable to analyse the long-term complications regarding mortality analysis, dislocations, periprosthetic fractures, and loosening of the prostheses. Furthermore, there was no comparative group of patients who had undergone surgery using an osteosynthesis approach. This study did not account for preexisting acetabular abnormalities, such as osteoarthritis, that may influence the clinical outcomes of hemiarthroplasty. A larger randomized trial with long-term follow-up is necessary to evaluate these issues.

## Conclusions

According to our study results, primary cemented bipolar hemiarthroplasty is a good choice for freely mobile osteoporotic elderly patients with unstable intertrochanteric femur fractures (AO/OTA type 31 A2 and A3). It enables early weight bearing, a shorter hospital stay, and a quick return to pre-fracture activities. The rehabilitation of patients is easy and fast, and none of them requires revision surgery in the first year. It reduces the potential complications of prolonged immobilization such as pressure sores, pulmonary complications, and deep vein thrombosis by early mobilization, thus reducing the family burden. Hemiarthroplasty is advantageous, but patients need to modify routine activities like squatting and sitting cross-legged on the floor. Future confirmation research is necessary to demonstrate the validity and reliability of our assumptions.
